# Effect of Irrigation to Winter Wheat on the Radiation Use Efficiency and Yield of Summer Maize in a Double Cropping System

**DOI:** 10.1100/2012/476272

**Published:** 2012-04-30

**Authors:** Li Quanqi, Chen Yuhai, Zhou Xunbo, Yu Songlie, Guo Changcheng

**Affiliations:** ^1^College of Water Conservancy and Civil Engineering, Shandong Agricultural University, Tai'an 271018, China; ^2^State Key Laboratory of Crop Biology, Shandong Key Laboratory of Crop Biology, Shandong Agricultural University, Tai'an 271018, China; ^3^Tianjin Key Laboratory of Water Resources and Environment, Tianjin Normal University, Tianjin 300074, China

## Abstract

In north China, double cropping of winter wheat and summer maize is a widely adopted agricultural practice, and irrigation is required to obtain a high yield from winter wheat, which results in rapid aquifer depletion. In this experiment conducted in 2001-2002, 2002-2003, and 2004-2005, we studied the effects of irrigation regimes during specific winter wheat growing stage with winter wheat and summer maize double cropping systems; we measured soil moisture before sowing (SMBS), the photosynthetic active radiation (PAR) capture ratio, grain yield, and the radiation use efficiency (RUE) of summer maize. During the winter wheat growing season, irrigation was applied at the jointing, heading, or milking stage, respectively. The results showed that increased amounts of irrigation and irrigation later in the winter wheat growing season improved SMBS for summer maize. The PAR capture ratio significantly (LSD, *P* < 0.05) increased with increased SMBS, primarily in the 3 spikes leaves. With improved SMBS, both the grain yield and RUE increased in all the treatments. These results indicate that winter wheat should be irrigated in later stages to achieve reasonable grain yield for both crops.

## 1. Introduction

In north China, the most important crops are winter wheat and summer maize; thus, a double cropping practice has been widely adopted [1, 2]. Evapotranspiration during the winter wheat growing season is approximately 400–500 mm, but annual precipitation typically does not exceeded 200 mm [3, 4]. Therefore, additional irrigation is required to achieve a satisfactory winter wheat grain yield. The summer maize growing season (June to September) is during the rainy season in north China. The average annual precipitation during the summer maize growing season is approximately 325 mm, which meets the crop's water consumption requirements. However, due to the influence of seasonal winds, drought often occurs during the summer maize seedling stage; therefore, soil moisture before sowing (SMBS) has become an important parameter to obtain a stable yield.

Previously, Li et al. [5] studied the winter wheat and summer maize double cropping system of winter wheat and summer maize in north China, including the influence of irrigation during the winter wheat growing season on water use and physiological characteristics of summer maize. The results showed that irrigation during the winter wheat growing season could increase soil moisture accumulation before summer maize sowing. With increased SMBS, the water use efficiency (WUE) of summer maize could increase in dry and moderate years, but not in wet years. Irrigation during the winter wheat growing season also affected the photosynthesis rate, transpiration, stomatal conductance, and leaf temperature of summer maize [6]. Thus, irrigation during the winter wheat growing season not only influenced the water consumption characteristics of winter wheat, but also the following summer maize crop. Given the excessive exploitation of groundwater resources for irrigation in north China [7–9], in a double cropping system, the combined grain yield and WUE for both winter wheat and summer maize should be considered.

Biomass production occurs when leaves intercept incoming photosynthetic active radiation (PAR) and the plant transforms the intercepted radiation into energy [10, 11]. Throughout the world, many researchers have studied the relationship between crop grain yield and RUE of crops [12–14]. To support agricultural water conservation efforts in north China, many researchers have studied the relationship between deficit irrigation and RUE. Li et al. [4] indicated that irrigating at the jointing and heading or jointing, heading, and milking stages could help increase the PAR capture ratio later in the winter wheat growing season. Han et al. [15] reported that varietal and deficit irrigation effects on the RUE and grain yield of winter wheat resulted from PAR modifications in the winter wheat canopies. Li et al. [16] showed that in north China, a furrow planting pattern should be used in combination with deficit irrigation to increase both the RUE and grain yield of winter wheat. However, all of these studies focused only on one crop, and did not examine deficit irrigation, RUE, and the planting system together.

This study aimed to determine whether irrigation regimes during specific winter wheat growing stages in a double cropping system affect summer maize leaf area characteristics responsible for intercepting incoming radiation and RUE.

## 2. Materials and Methods

### 2.1. Experimental Site

The experiments were conducted using 12 irrigation plots at Yucheng Comprehensive Experimental Station (36°57′N, 116°38′E), Chinese Academy of Science, during the years 2001-2002 and 2002-2003, and at Tai'an Experimental Station (36°10′N, 117°09′E), Agronomy College, Shandong Agricultural University, in 2004-2005. The plot areas at Yucheng and Tai'an were 6.7 and 9.0 m^2^, respectively, at a depth of 1.5 m; the plots were enclosed with a concrete wall, and the bottom surfaces of the plots were not sealed. The plot surfaces were 15 cm above ground level on all sides to prevent runoff, run-on, and subsurface water movement between the plots. The 2 experimental stations are located in Shandong province, north China. The mean annual precipitation at the Yucheng Comprehensive Experimental Station is 590 mm, of which approximately 62% falls between June and September—the summer maize growing season. The soil at the experimental site is classified as sandy loam with an organic matter content of approximately 0.5–0.6%, pH of approximately 8.5, and field capacity and wilting point of 25.1% and 8.0% by volume, respectively. The mean annual precipitation at the Tai'an Experimental Station is 700 mm, of which approximately 65% falls between June and September. The soil at this experimental site is classified as loam with an organic matter content of approximately 1.4%, pH of approximately 6.9, and field capacity and wilting point of 25.8% and 7.7% by volume, respectively. The precipitation under natural conditions for the summer maize growing seasons in 2002, 2003, and 2005 were 133.6, 308.4, and 576.4 mm, respectively. Base on annual mean precipitation during the summer maize growing season in the studied regions, the year 2002 was a very dry year, 2003 was a moderate year, and 2005 was a wet year.

### 2.2. Experimental Design

The experiments were conducted in triplicate using a randomized block design during 2001-2002, 2002-2003, and 2004-2005; the following 4 irrigation amounts were applied throughout the entire growth cycle of winter wheat: no supplemental irrigation (T0); irrigated only at the jointing stage (T1); irrigated at the jointing and heading stages (T2); irrigated at the jointing, heading, and milking stages (T3). In 2002, irrigation was applied on March 23, April 12, and May 9; in 2003, on April 6, April 30, and May 16; in 2005, on April 7, April 27, and May 14. All irrigation applications consisted of 60 mm of water supplied from a pump outlet to the plots via plastic pipes; a flow meter was used to measure the amount of water applied.

### 2.3. Cultural Procedures and Measurements

Winter wheat was planted on October 4 in 2001 and 2002, and on October 6 in 2004. Before sowing, 300 kg·hm^−2^ of triple superphosphate, 300 kg·hm^−2^ of urea, and 75 kg·hm^−2^ of potassium chloride were applied. The wheat plants were harvested on June 6 in 2002 and 2003, and June 7 in 2005. The maize cultivar “Nongda108” was manually planted immediately after harvesting in all 3 years. At the beginning of July, urea was applied at a rate of 140 kg·hm^−2^ depending on the rainfall. When the maize plants were at the 5-leaf stage, their density was fixed at 6.6 × 10^4^ plants·hm^−2^. The maize plants were harvested on September 24, September 26, and September 28 in 2002, 2003, and 2005, respectively. After air-drying, the dry weight of the grain was measured.

Beginning at 28 days after sowing, the leaf area index (LAI) was measured every 7 days using a LI-3000 Portable Area Meter (Li-Cor Co.Ltd, Lincoln, Nebraska, USA) at Yucheng Comprehensive Experimental Station in 2002 and 2003. At the Agronomy Station of Shandong Agricultural University, the LAI was measured using a 1.5 m long linear sensor (SunScan) every 10 days from emergence to maturity.

To measure transmitted radiation, the linear sensor (SunScan) was placed parallel to the row direction of each plot in the middle of each summer maize row. The average of these measurements was considered to be the radiation transmitted by the canopy. Moreover, the transmitted radiation at the 3 spike leaves (including the leaves on, above, and below the spike), above the 3 spike leaves, and below the 3 spike leaves were measured, respectively. The incoming solar radiation above the crop canopy was also monitored. We determined the amount of solar radiation intercepted by the canopy by calculating the difference between the above-canopy and soil surface solar radiation as measured by the SunScan [16].

RUE during the summer maize growing season was calculated using the approach proposed by Quanqi et al. [17]:

(1) $$E\%=\frac{\Delta W\cdot H}{\Sigma S}\times 100\%,$$

where Δ*W* is dry matter weight at each growing stage after drying at 80°C for constant weight; *H* = 17.782 KJ·g^−1^ is the energy conversion coefficient; and Σ*S* is global incoming radiation for each summer maize growing season, which were obtained from weather stations located near the experiment area at Yucheng and within 0.5 km of the experimental site at Tai'an.

### 2.4. Statistical Analysis

The treatments were run as an analysis of variance (ANOVA). For ANOVA, *α* = 0.05 was set as the level of significance to determine whether differences existed among treatments means. The multiple comparisons were done for significant effects with the least significant difference (LSD) test at *α* = 0.05.

## 3. Results

### 3.1. Soil Moisture Accumulation before Summer Maize Sowing

Irrigation during the winter wheat growing season has the effect of increasing the soil moisture accumulation before summer maize sowing (Table 1). With more irrigation, soil moisture in the 1.2 m soil profiles increased significantly (LSD, *P* < 0.05), by 10.5, 42.3, and 75.4 mm at T1, T2, and T3, respectively, compared to T0 in 2002: by 23.3, 31.2, and 61.4 mm, respectively, in 2003; by 19.5, 68.2, and 102.1 mm, respectively, in 2005. The average soil moisture after the first, second, and third irrigations improved by 17.7, 47.2, and 79.6 mm, respectively. These results indicated that irrigation later in the winter wheat growing season and increased irrigation amounts improved SMBS for summer maize.

**Figure 1:**
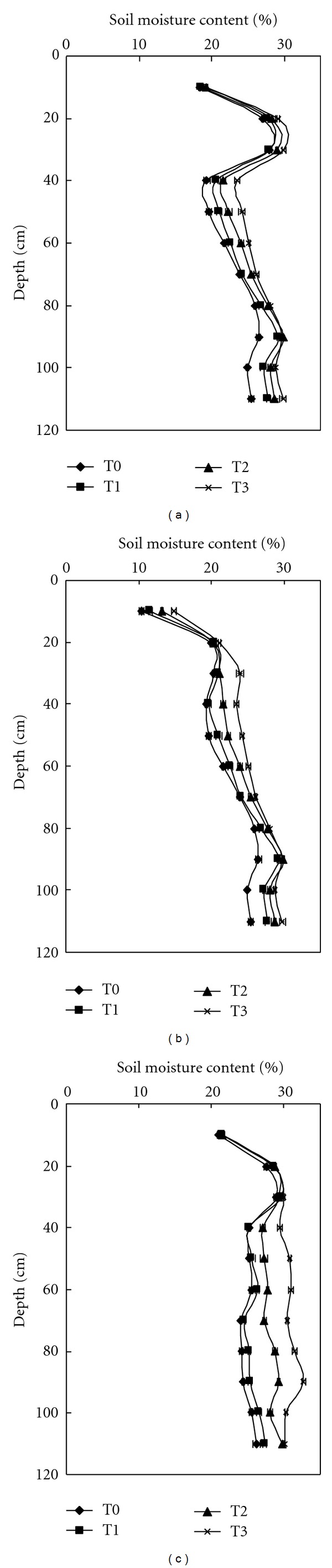
Soil moisture status before summer maize sowing in 2002 (a), 2003 (b), and 2005 (c). Horizontal bars are standard errors.

However, the effect of irrigation at each soil layer was different; soil moisture was increased significantly (LSD, *P* < 0.05) for the soil layer below 20 cm, but not above it (Figure 1).

### 3.2. Leaf Area Index

**Figure 2:**
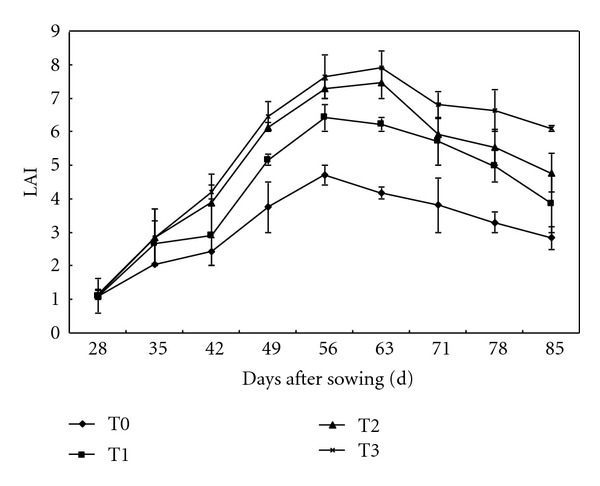
The dynamic variation of LAI in 2002 at Yucheng. Vertical bars are standard errors.

Figure 2 shows dynamic LAI variations during the summer maize growing season in 2002. The values corresponding to 2003 and 2005 are not shown because they are very similar to the values shown for 2002. As shown in Figure 2, LAI increased with growth stages. At approximately 56 days after sowing, LAI for each treatment group reached the maximum value. After that point, LAI decreased as the growing season continued. During the summer maize growing season, LAI was highest at T3, with a maximum value of 7.9, which was higher than those at T2, T1, and T0 by 3.2, 1.7, and 0.5, respectively. For the summer maize growing season overall, LAI at T3 was higher than at other treatments, which could have significantly affected the complete capture and utilization of PAR.

### 3.3. PAR Capture Ratio

Table 2 shows the PAR reflection ratio, PAR penetration ratio, and PAR capture ratio later in the summer maize growing season. The PAR reflection ratio was not significantly (LSD, *P* < 0.05) different at T0, T1, and T2, all of which were significantly (LSD, *P* < 0.05) higher than at T3. The PAR penetration ratio and PAR capture ratio were significantly (LSD, *P* < 0.05) different between treatments. With increased summer maize SMBS, the PAR penetration ratio significantly (LSD, *P* < 0.05) decreased for all treatment groups; however, the PAR capture ratio significantly (LSD, *P* < 0.05) increased, primarily due to improved LAIs associated with the increased SMBS (Figure 2).

The PAR capture ratios at the 3 spike leaves were contrary to those obtained below the 3 spike leaves (Table 3). However, the PAR capture ratios below the 3 spike leaves were consistent with the PAR penetration ratios, that is, more PAR captured below the 3 spike leaves resulted in more PAR penetration. The PAR capture ratio at T3 was higher than at T2, T1, and T0 by 3.2%, 5.2%, and 9.6%, respectively. However, at the 3 spike leaves, the PAR capture ratio at T3 was higher than at T2, T1, and T0 by 4.1%, 10.6%, and 24.9%, respectively. Thus, although irrigation during the winter wheat growing season clearly altered the LAI, it had little effect on the PAR interception amount; however, it had significantly (LSD, *P* < 0.05) affected the vertical distribution of PAR in the canopy. With increased SMBS, the PAR capture ratio in the 3 spike leaves improved.

### 3.4. Dry Matter Accumulation at Maturity and Grain Yield

Table 4 shows the dry matter accumulation at maturity and grain yield for summer maize. The average dry matter accumulation at maturity at T0 was 14587.2 kg·hm^−2^, which was significantly (LSD, *P* < 0.05) lower than at T2 and T3 by 303.2 and 316.3 kg·hm^−2^, respectively. The average grain yield at T0 was only 6710.1 kg·hm^−2^, which was significantly (LSD, *P* < 0.05) lower than at T2 and T3 by 586.2 and 890.7 kg·hm^−2^, respectively. Thus, it is apparent that with increased SMBS, the dry matter accumulation and grain yield also increase consistently.

### 3.5. Radiation Use Efficiency

Table 5 shows the RUE of summer maize at different growth stages. During the summer maize growing season, the highest RUE was observed at the milking stage. At jointing, large bell-mouth, tasseling, milking, and maturity stages, the RUE at T3 was significantly (LSD, *P* < 0.05) higher than at T0. Except at the jointing stage, the RUE at T3 was not significantly (LSD, *P* < 0.05) different than at T2. Furthermore, the RUE at T0 was lower than at T1, T2, and T3 by 4.5%, 9.1%, and 18.2%, respectively. Hence, in a winter wheat and summer maize double cropping system, RUE was enhanced during the summer maize growing season by increased irrigation amounts and irrigation later in the winter wheat growing season.

## 4. Discussion

In north China, approximately 70% of the total cultivated land is planted with winter wheat and summer maize in a double cropping system [18]. The experiment showed that the timing and amount of irrigation during the winter wheat growing season could affect the SMBS for summer maize in a double cropping system. Although the irrigation water applied during the winter wheat growing season was not absorbed completely, some of it was utilized by the summer maize crop. The results indicated that irrigation later in the winter wheat growing season and increased irrigation amounts improved SMBS for summer maize. With increased SMBS, the grain yield and RUE for summer maize improved consistently. Former studies by the authors [3] showed that during the winter wheat growing season in north China, irrigation applied at the jointing and heading stages or jointing and milking stages had no significantly (LSD, *P* < 0.05) differences between the grain yield and WUE for winter wheat. Therefore, by adopting effective measures, such as irrigation at the jointing and milking stages during the winter wheat growing season, satisfactory grain yields for winter wheat and summer maize can be obtained in a double cropping system. The experiment also showed that in winter wheat and summer maize double cropping systems, research studies that focused only on winter wheat water consumption was not sufficient. Researchers should consider the effect of winter wheat irrigation regimes on the grain yield, RUE, WUE, and economic benefits of the entire planting system. Similarly, in order to conserve agricultural water in north China, irrigation regimes should be customized based on specific planting systems.

Most of the green organ photosynthetic matter was produced by the 3 spike leaves [19], hence, an increase in the green organ's PAR capture ratio would aid in the accumulation and transportation of photosynthetic products later in the summer maize growing season. Therefore, the improved PAR capture ratio and transformational ability in these plant parts were very important for increasing the quantity of photosynthetic matter. The results presented in this experiment show that the PAR capture ratio associated with the high yielding treatment (T3) at the 3 spike leaves was 54.3%. This result is consistent with Fang's study of winter wheat [20]. It is apparent that increasing SMBS could optimize PAR distributions in the summer maize canopy, which will result in a highly efficient photosynthetic colony.

The amount of incoming PAR that is absorbed by the canopy primarily depends on LAI and crop geometry [21]. The differences in the PAR capture ratios for the summer maize canopies were not only due to dynamic LAI variations, but also due to alterations in vertical distributions. Although vertical LAI distributions were not included in this paper, the topic will be addressed in a future study.

## Figures and Tables

**Table 1:** Soil moisture accumulation in 0–1.2 m soil profiles before summer maize sowing (mm).

Treatments	2002	2003	2005	Mean
T0	221.4^c^	194.0^d^	247.2^d^	220.9^d^
T1	231.9^c^	217.3^c^	266.7^c^	238.6^c^
T2	263.7^b^	225.2^b^	315.4^b^	268.1^b^
T3	296.8^a^	255.4^a^	349.3^a^	300.5^a^

Means in each column with in each year followed by the same letter are not significantly different at *P* < 0.05 based on LSD test.

**Table 2:** Effect of irrigation during the winter wheat growing season on the PAR reflection ratio, PAR penetration ratio, and PAR capture ratio in summer maize canopy (%).

Treatments	PAR reflection ratio	PAR penetration ratio	PAR capture ratio
T0	3.8^a^	20.4^a^	75.8^d^
T1	3.7^a^	16.1^b^	80.2^c^
T2	3.3^a b^	14.5^c^	82.2^b^
T3	2.9^c^	11.7^d^	85.4^a^

The data was the average values on August 20, Aug 27, and Septemper 5, 2002; and Aug 24, and Sep 9, 2003; and Aug 19, Aug 20, and Aug 22, 2005. Values followed by a different letter are significantly different at 5% probability level.

**Table 3:** PAR capture ratio in the canopy of summer maize (%).

Treatment	Above the 3 spike leaves	The 3 spike leaves	Below the 3 spike leaves
T0	12.8^d^	29.4^d^	33.6^a^
T1	15.1^c^	43.7^c^	21.4^b^
T2	20.6^b^	50.2^b^	11.4^c^
T3	22.0^a^	54.3^a^	9.1^d^

The data was the average values on Aug 20, Aug 27, and Sep 5, 2002; and Aug 24, and Sep 9, 2003, and Aug 19, Aug 20, and Aug 22, 2005. Values followed by a different letter are significantly different at 5% probability level.

**Table 4:** Effect of irrigation during the winter wheat growing season on the dry matter accumulation at maturity and grain yield of summer maize (kg · hm^−2^).

Treatment	Dry matter accumulation	Grain yield
T0	14587.2^b^	6710.1^c^
T1	14616.7^ab^	7016.0^bc^
T2	14890.4^a^	7296.3^ab^
T3	14903.5^a^	7600.8^a^

The data was the average values in 2002, 2003, and 2005. Values followed by a different letter are significantly different at 5% probability level.

**Table 5:** Effect of irrigation during the winter wheat growing season on the RUE of summer maize at different growth stages (%).

Treatment	Jointing	Large bell-mouth	Tasseling	Milking	Maturity
T0	0.6^d^	1.1^b^	3.1^c^	3.7^b^	2.2^b^
T1	0.7^c^	1.2^b^	3.2^bc^	3.9^ab^	2.3^b^
T2	0.8^b^	1.3^ab^	3.4^ab^	4.0^ab^	2.4^ab^
T3	0.9^a^	1.5^a^	3.6^a^	4.1^a^	2.6^a^

The data was the average values in 2002, 2003, and 2005. Values followed by a different letter are significantly different at 5% probability level.
